# Supplementary material for: The Effect of Curcumin on Reducing Atherogenic Risks in Obese Patients with Type 2 Diabetes: A Randomized Controlled Trial
Source: Nutrients. 2024 Jul 26;16(15):2441. doi: 10.3390/nu16152441 (PMC11314193; doi:10.3390/nu16152441)
Supplement: Supplementary file 1 [file nutrients-16-02441-s001.zip › nutrients-3086459-supplementary.pdf]

Supplementary material

Table S1. Mean Daily Intake of Nutrients by Subjects at Baseline and 12 Months in Placebo and Curcumin Groups

**Daily intake of nutrients**	**Placebo (n=114)**		**Curcumin (n=113)**		***P*[2]**
	**Baseline[1]**	**12 months**	**Baseline[1]**	**12 months**	
Energy (kcal/d)	1857.60±110.97	1893.74±74.30	1864.21±87.98	1881.16±67.23	0.100
Carbohydrate (%energy)	57.50±2.57	58.07±2.56	57.04±1.52	58.08±1.62	0.148
Protein (%energy)	12.98±2.13	13.21±1.35	13.33±1.28	13.44±1.20	0.418
FAT (%energy)	28.27±2.12	28.91±1.91	28.47±2.42	28.44±2.23	0.056
Fiber (g/d)	8.54±1.16	8.46±0.88	8.49±0.82	8.39±0.64	0.464

[1] All parameters are presented as mean ± SD. There are no significant differences between the two groups at baseline for any variable, as determined by *t* tests.

[2] Curcumin had no significant effect on mean daily intake of nutrients, as assessed by one-factor ANCOVA with the baseline value as a covariate.

There were no significant differences in mean daily energy intake (energy, carbohydrate, protein, fat, and fiber) and nutrient intakes between the curcumin and placebo groups.

Figure S1. CONSORT Flow Diagram of Patient Enrollment, Allocation, Follow-Up, and Analysis

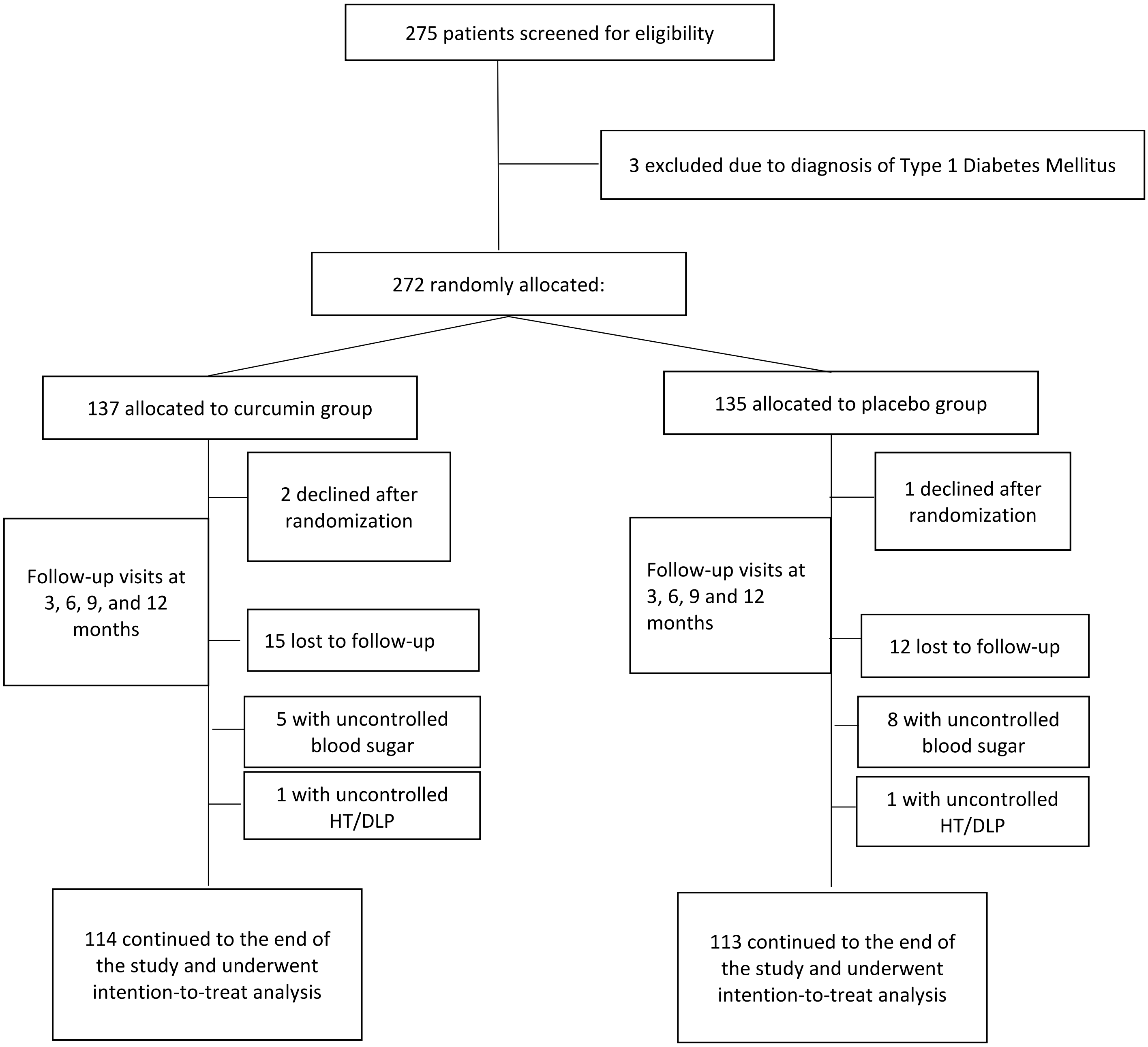

Figure S2. Fingerprints of Curcuminoid Extracts

The high-performance thin layer chromatography (HPTLC) chromatogram of GPO curcuminoid extract is shown in Figure 2, compared with the HPTLC chromatogram of the curcumin (curcuminoids) standard in Figure 1. In each batch of GPO curcuminoid extract, the peak ratio of curcumin to demethoxycurcumin to bisdemethoxycurcumin is controlled to be 1: not more than 0.6: not more than 0.4.

Calibration: M: CURMIN — No name
Method: Scan Integration — Calibration Data END
Analysis a:

Peak	Height	Area
1	12.1	401.8
2	29.2	1385.1
3	61.1	1878.2

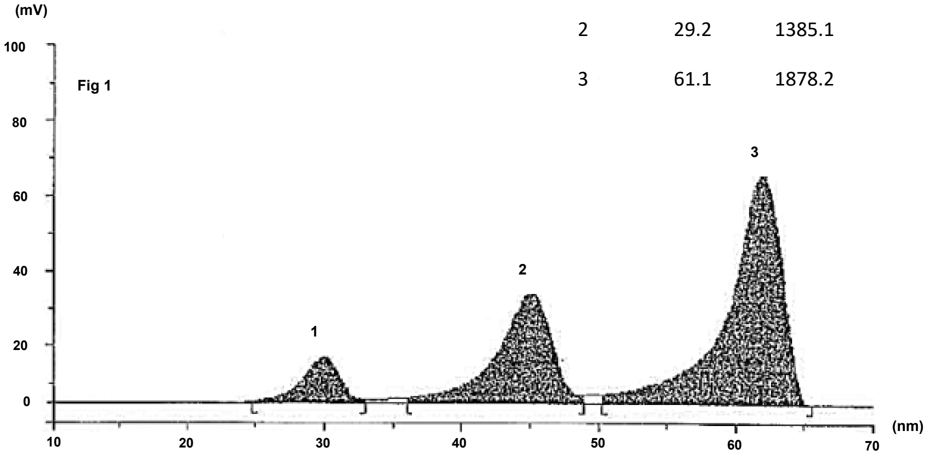

Wavelength: 420 nm

Track: 1, noise level: 0.019 mV, raw data file: 061011

Calibration: M: CURMIN — No name
Method: Scan Integration — Calibration Data END
Analysis a:

Peak	Height	Area
1	18.2	620.7
2	32.2	1528.7
3	68.5	3301.2

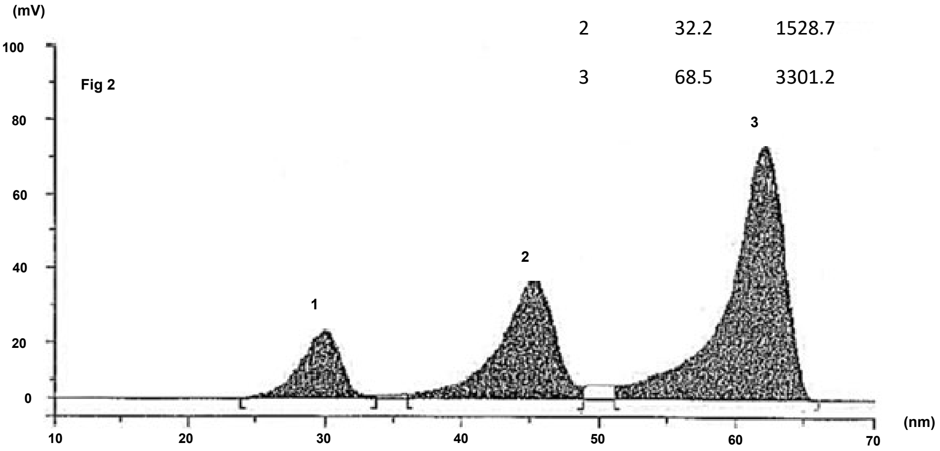

Wavelength: 420 nm

Track: 2, noise level: 0.019 mV, raw data file: 061011

Table S2. Number of Capsules Consumed by Subjects per 3 Months and per Day, Counted at the 3-, 6-, 9-, and 12-Month Visits

		Placebo		Curcumin		*P-value*
		Number of subjects who showed up	Number of capsules taken (mean, SE)	Number of subjects who showed up	Number of capsules taken (mean, SE)	
Consumption per 3 months	3-month visit	114	558.69 (54.59)	113	559.13 (52.11)	0.58
	6-month visit	114	558.55 (21.61)	113	556.72 (25.61)	0.13
	9-month visit	114	517.16 (20.32)	113	514.96 (21.33)	0.43
	12-month visit	114	515.06 (20.82)	113	514.11 (21.78)	0.33
Consumption per day	3-month visit	114	6.21 (0.60)	113	6.21 (0.58)	0.57
	6-month visit	114	6.20 (0.23)	113	6.19 (0.28)	0.13
	9-month visit	114	5.70 (0.22)	113	5.72 (0.24)	0.43
	12-month visit	114	5.65 (0.21)	113	5.85 (0.25)	0.33

Table S3. Adverse Effects (Creatinine, AST, ALT) in Curcumin-Treated and Placebo-Treated Groups at Each Follow-Up Visit

Variables	Visits	Placebo		Curcumin		*P*
		Mean (SE)	Min-Max	Mean (SE)	Min-Max	
Creatinine (mg/dL)	Baseline	0.87 (0.02)	0.40-1.69	0.86 (0.02)	0.45-1.6	0.77
	3 months	0.88 (0.02)	0.45-1.81	0.91 (0.05)	0.46-7.26	0.64
	6 months	0.94 (0.02)	0.47-2.04	0.92 (0.02)	0.52-1.70	0.40
	9 months	0.94 (0.02)	0.44-1.83	0.93 (0.02)	0.54-1.81	0.51
	12 months	0.87 (0.02)	0.40-1.69	0.85 (0.02)	0.45-1.60	0.77
Aspartate aminotransferase AST (U/L)	Baseline	25.01 (0.87)	11-89	25.34 (0.80)	13-67	0.58
	3 months	22.45 (0.79)	9-78	23.85 (0.90)	11-111	0.076
	6 months	23.53 (1.43)	8-214	24.12 (0.98)	10-89	0.47
	9 months	21.78 (0.63)	11-76	24.29 (1.23)	12-114	0.88
	12 months	25.01 (0.87)	11-89	25.41 (0.81)	13-67	0.54
Alanine aminotransferase ALT (U/L)	Baseline	27.58 (1.56)	5-145	30.09 (1.50)	5-118	0.08
	3 months	24.08 (1.1)	6-101	27.49 (1.7)	6-214	0.08
	6 months	24.74 (1.15)	7-98	28.16 (1.64)	7-186	0.18
	9 months	23.01 (1.14)	6-117	27.50 (1.80)	6-129	0.36
	12 months	27.58 (1.56)	5-145	30.27 (1.51)	8-118	0.21

Figure S3. Mean of parameters with SEM at baseline, 3, 6, 9 and 12 months were compared between placebo- and curcumin-treated group.

A

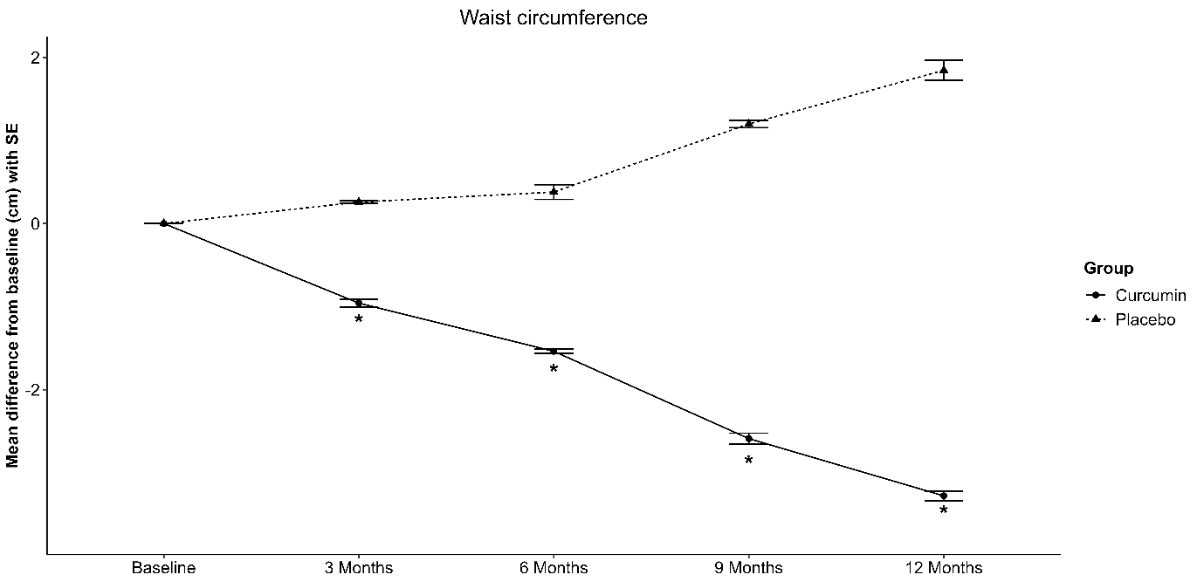

B

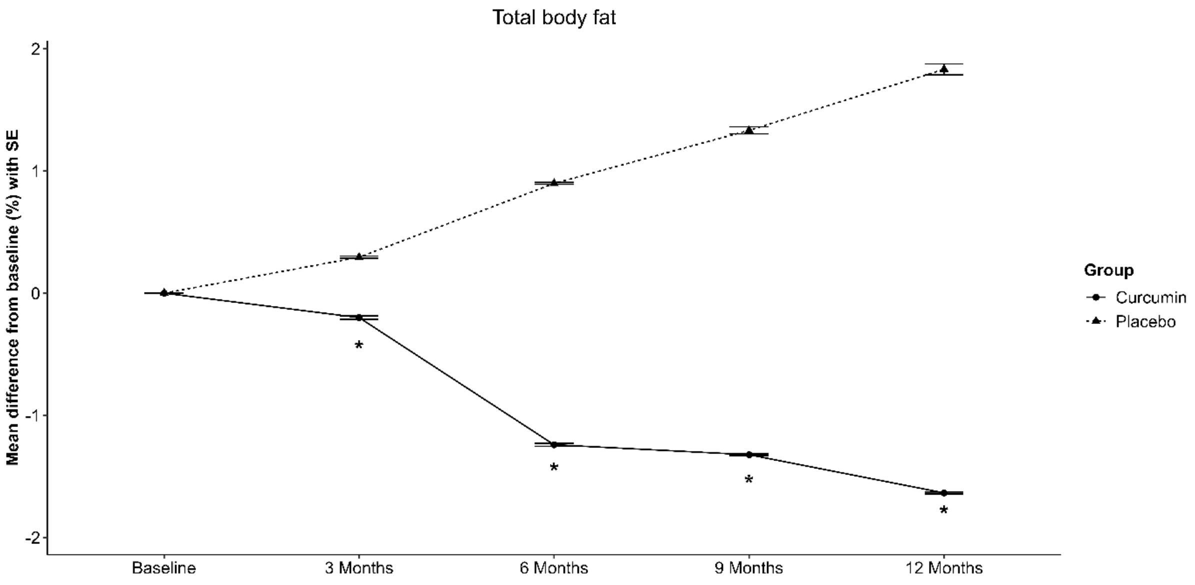

C

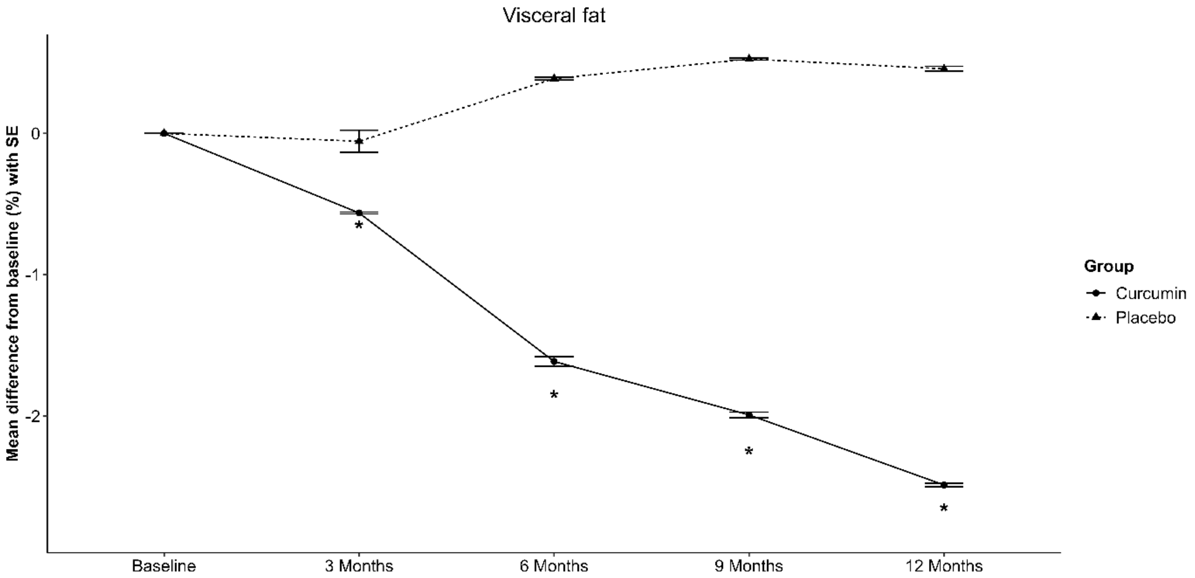
Visceral fat
Mean difference from baseline (%) with SE
0
-1
-2
Baseline
3 Months
6 Months
9 Months
12 Months
*
*
*
*
Group
Curcumin
Placebo

D

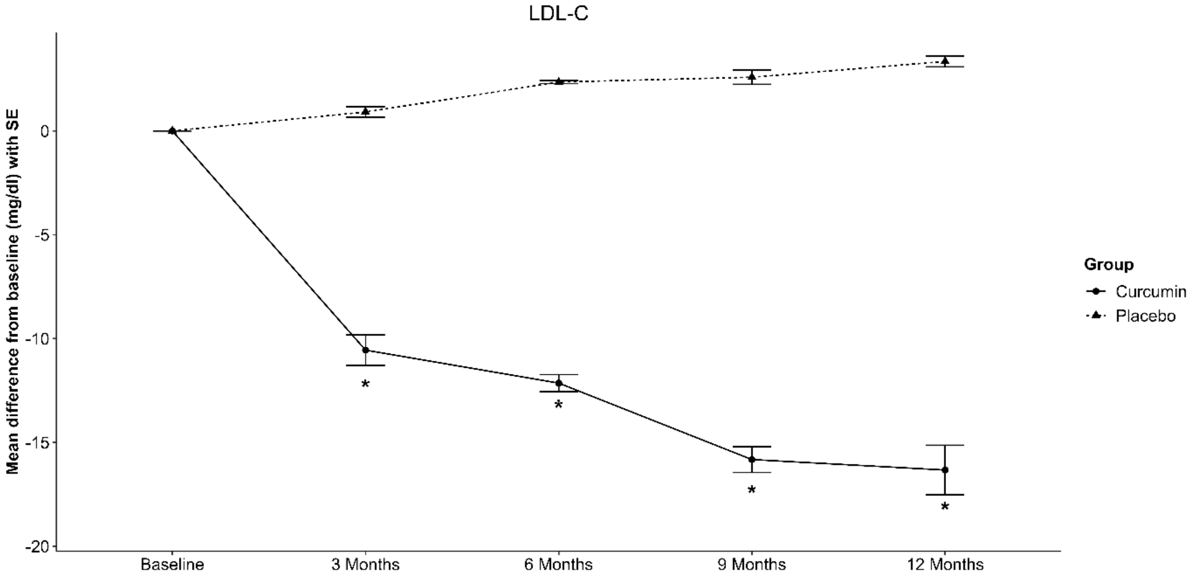
LDL-C
Mean difference from baseline (mg/dl) with SE
0
-5
-10
-15
-20
Baseline
3 Months
6 Months
9 Months
12 Months
*
*
*
*
Group
Curcumin
Placebo

E

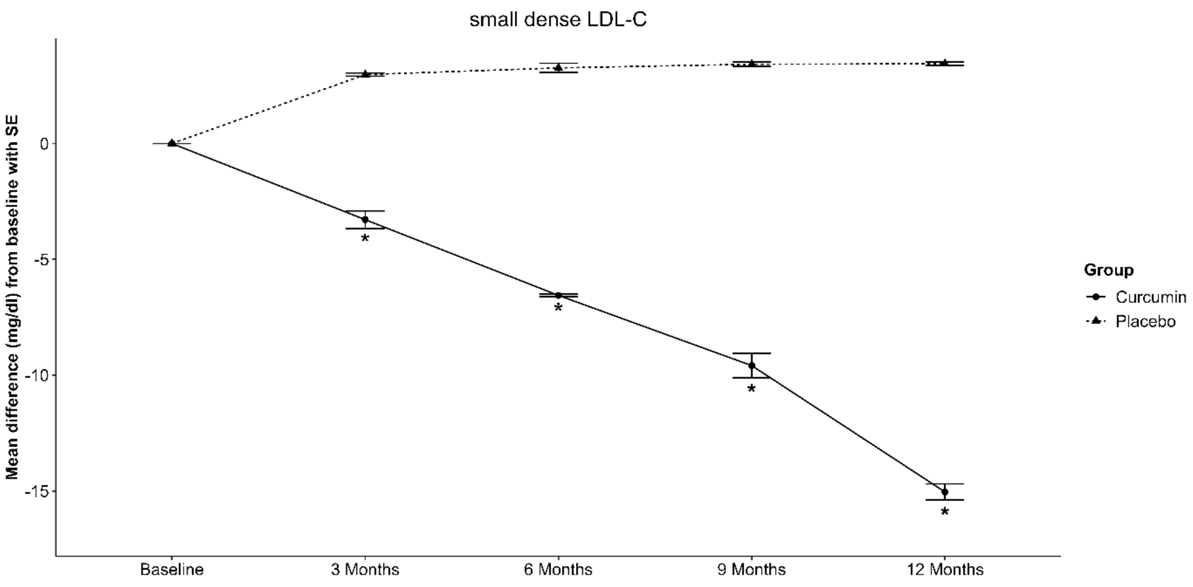
small dense LDL-C
Mean difference (mg/dl) from baseline with SE
0
-5
-10
-15
Baseline
3 Months
6 Months
9 Months
12 Months
Group
Curcumin
Placebo

F

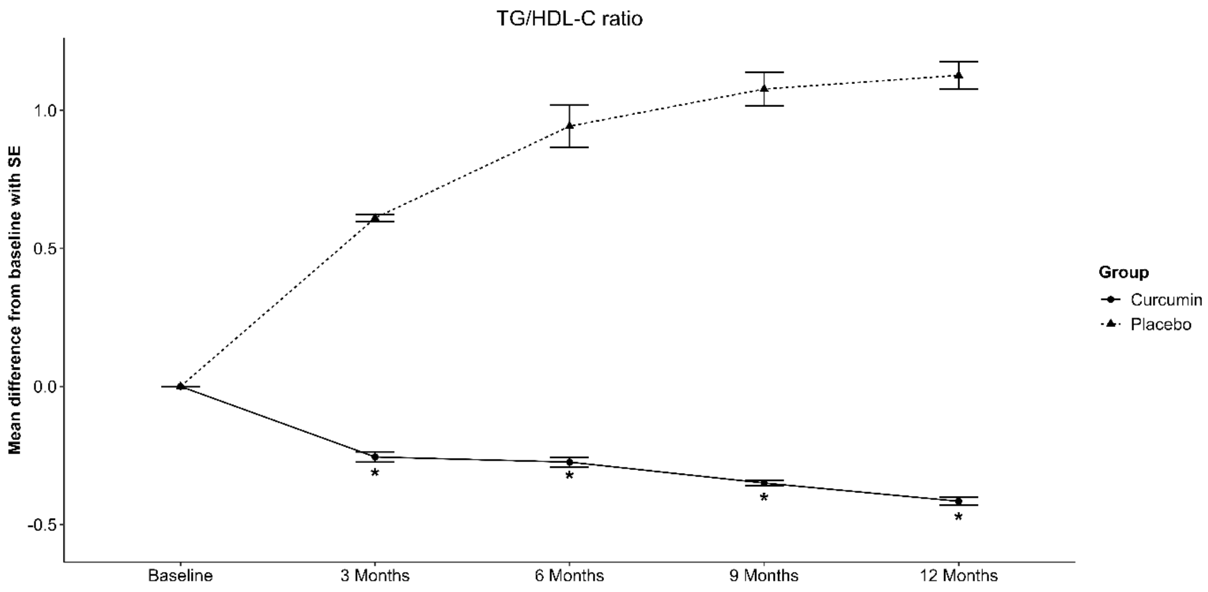
TG/HDL-C ratio
Mean difference from baseline with SE
1.0
0.5
0.0
-0.5
Baseline
3 Months
6 Months
9 Months
12 Months
Group
Curcumin
Placebo

K

G

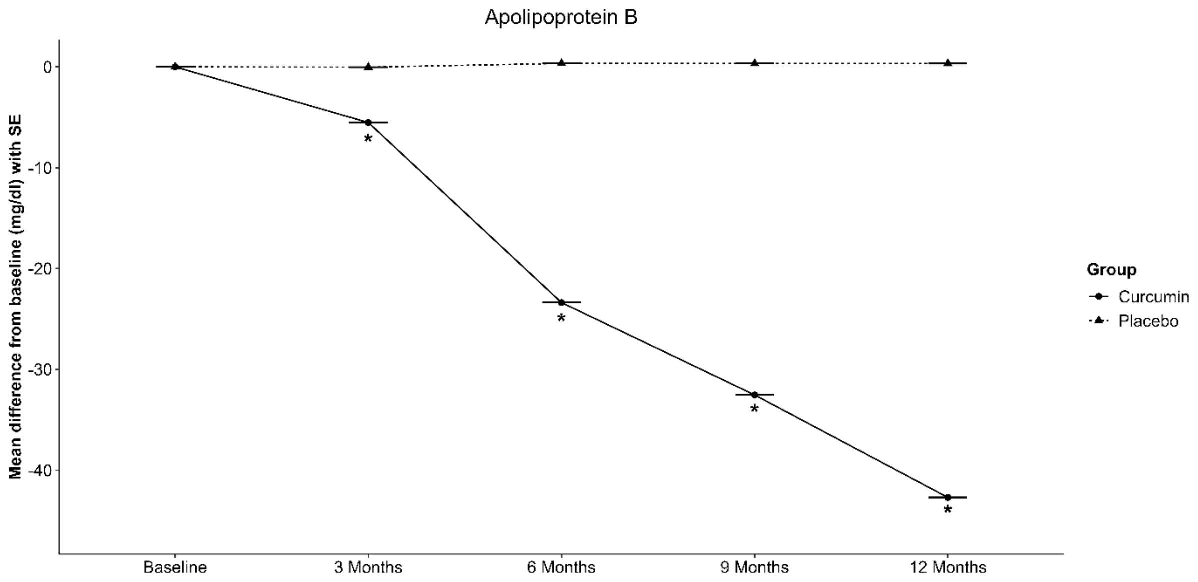

Apolipoprotein B
Mean difference from baseline (mg/dl) with SE
0
-10
-20
-30
-40
Baseline
3 Months
6 Months
9 Months
12 Months
Group
Curcumin
Placebo

H

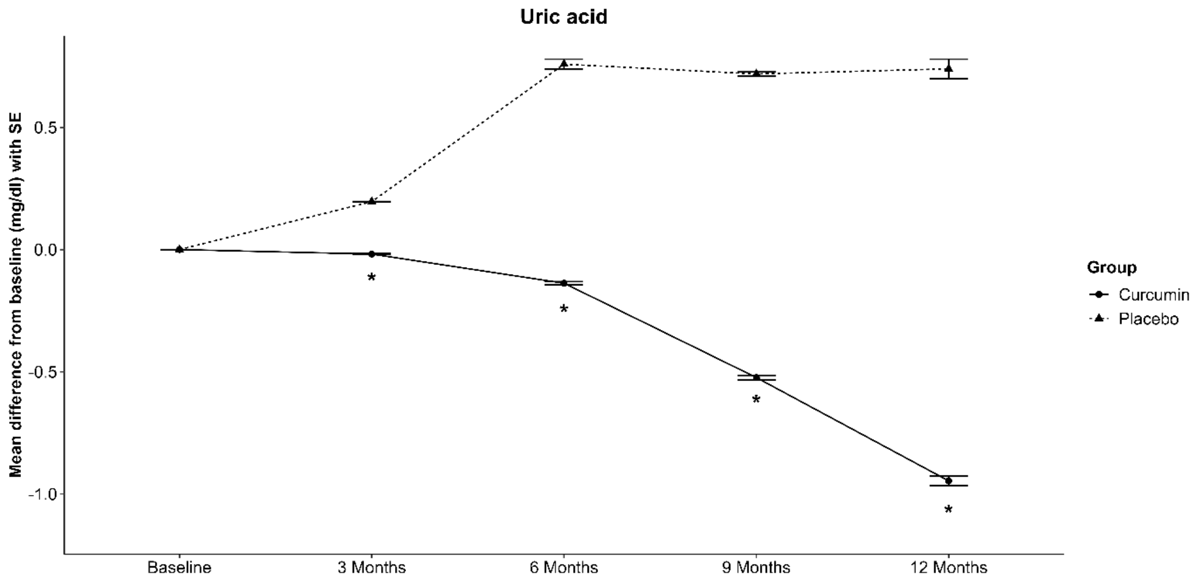

Uric acid
Mean difference from baseline (mg/dl) with SE
0.5
0.0
-0.5
-1.0
Baseline
3 Months
6 Months
9 Months
12 Months
Group
Curcumin
Placebo

I

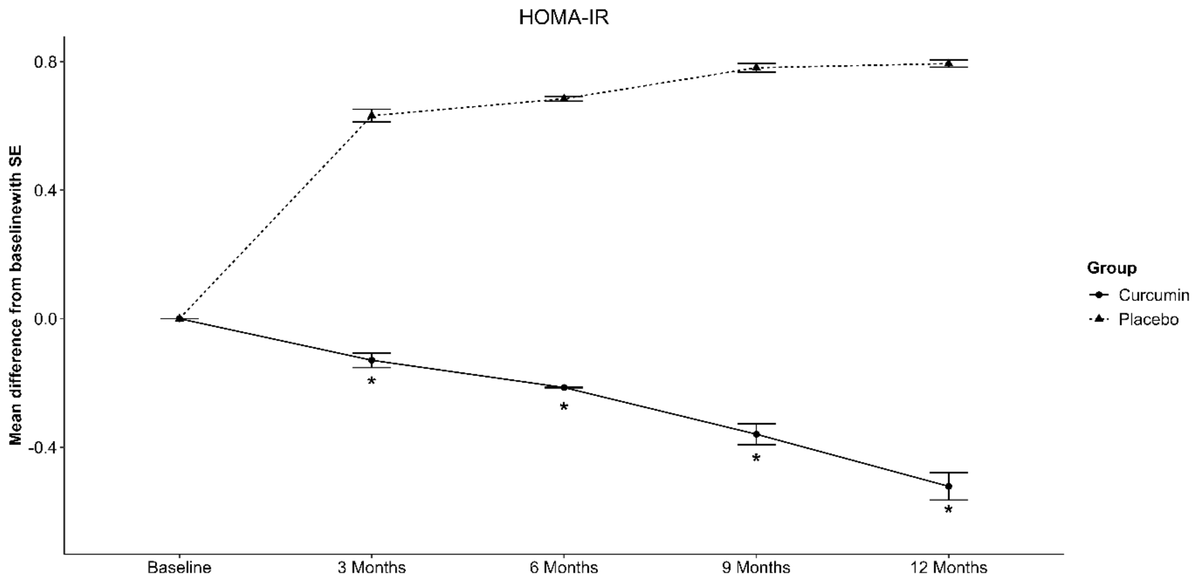
HOMA-IR
Mean difference from baselinewith SE
0.8
0.4
0.0
-0.4
Baseline
3 Months
6 Months
9 Months
12 Months
Group
Curcumin
Placebo
*
*
*
*

J

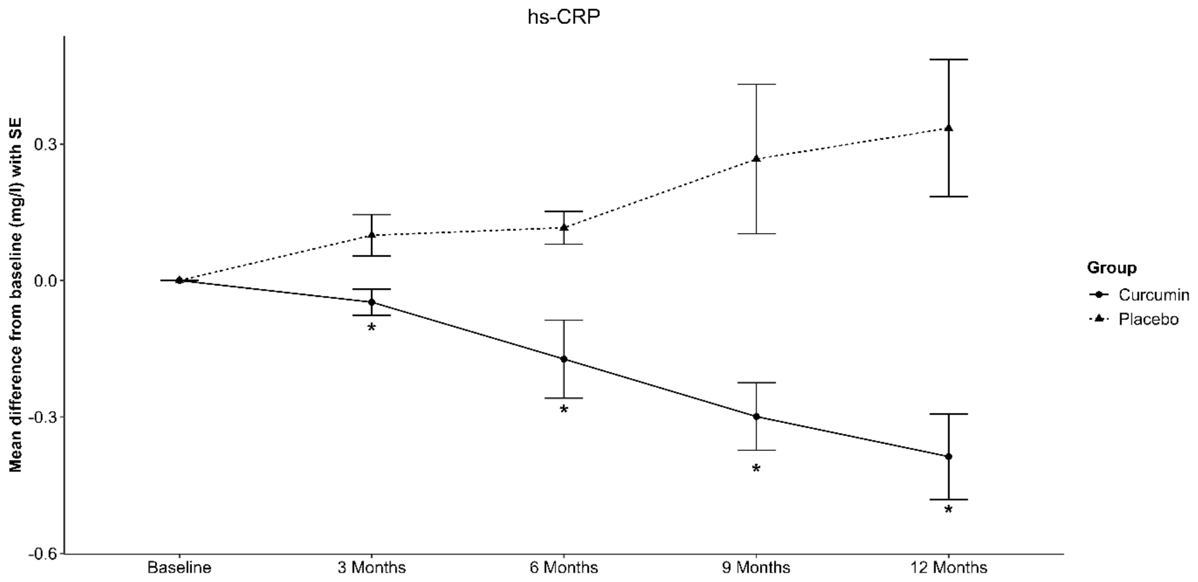
hs-CRP
Mean difference from baseline (mg/l) with SE
0.3
0.0
-0.3
-0.6
Baseline
3 Months
6 Months
9 Months
12 Months
Group
Curcumin
Placebo
*
*
*
*

K

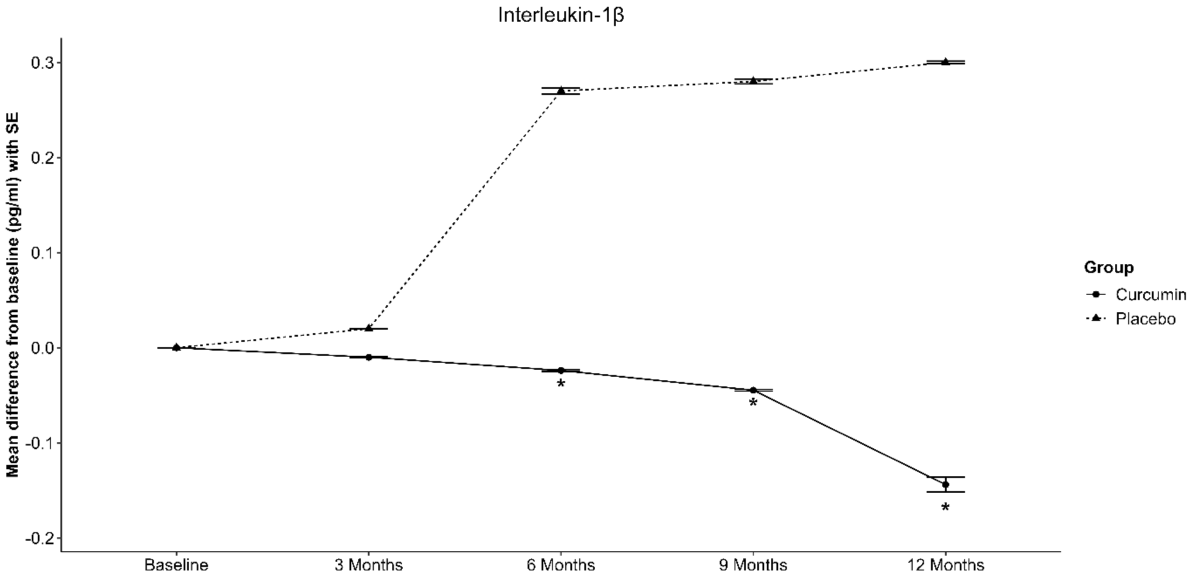
Interleukin-1β
Mean difference from baseline (pg/ml) with SE
0.3
0.2
0.1
0.0
-0.1
-0.2
Baseline
3 Months
6 Months
9 Months
12 Months
Group
Curcumin
Placebo
*
*
*

L

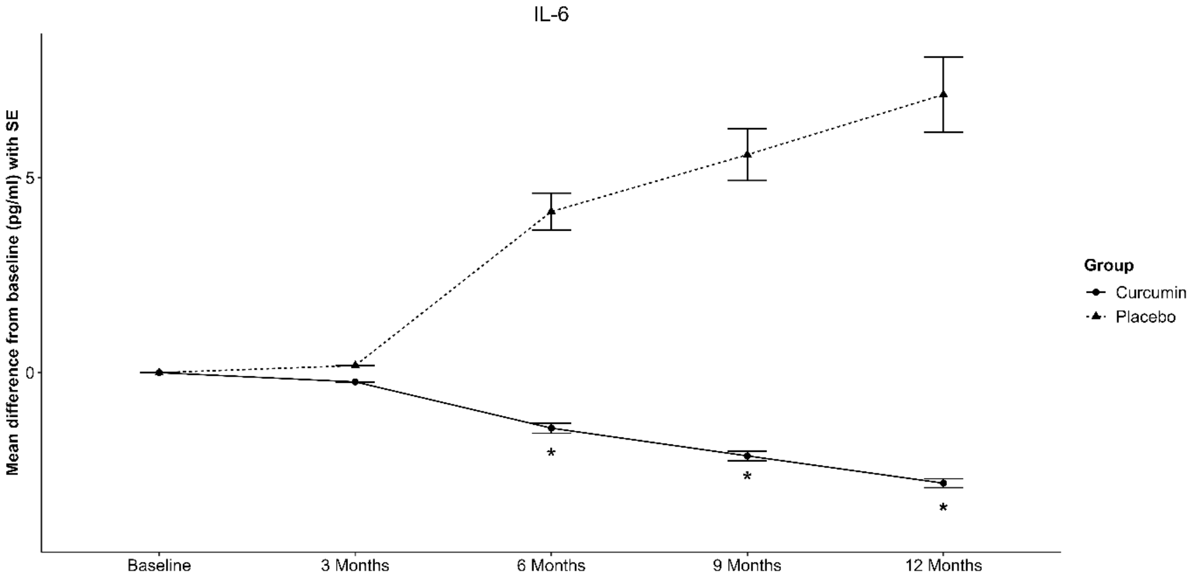
IL-6
Mean difference from baseline (pg/ml) with SE
5
0
Baseline
3 Months
6 Months
9 Months
12 Months
Group
Curcumin
Placebo
*
*
*

M

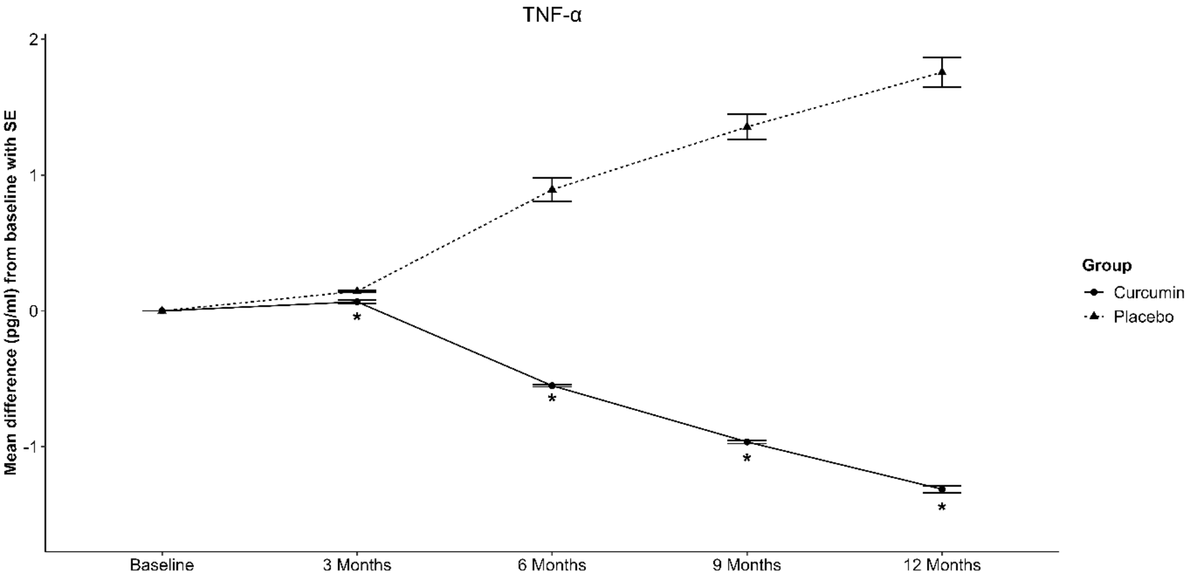

Figure S3. Mean of parameters with SEM at baseline, 3, 6, 9 and 12 months were compared between placebo- and curcumin-treated group. (A) Waist circumference; (B) Total body fat; (C) Visceral fat; (D) Low density lipoprotein cholesterol (LDL-C); (E) Small dense low density lipoprotein cholesterol (small dense LDL-C); (F) Triglyceride/High density lipoprotein ratio (TG/HDL ratio); (G) Apolipoprotein B; (H) Uric acid; (I) Homeostatic Model Assessment of Insulin Resistance (HOMA-IR); (J) high-sensitivity C-reactive protein (hs-CRP); (K) Interleukin -1β (IL-1β); (L); Interleukin -6 (IL-6); (M) Tumor necrosis factor alpha (TNF-α).
* Statistically significant
